# Missing the point: are journals using the ideal number of decimal places?

**DOI:** 10.12688/f1000research.14488.3

**Published:** 2018-08-10

**Authors:** Adrian G Barnett

**Affiliations:** 1 Institute of Health and Biomedical Innovation, School of Public Health and Social Work, Queensland University of Technology, 60 Musk Avenue, Kelvin Grove, Queensland, 4059, Australia

**Keywords:** decimal places, meta-research, readability, statistics

## Abstract

**Background**: The scientific literature is growing in volume and reducing in readability. Poorly presented numbers decrease readability by either fatiguing the reader with too many decimal places, or confusing the reader by not using enough decimal places, and so making it difficult to comprehend differences between numbers. There are guidelines for the ideal number of decimal places, and in this paper I examine how often percents meet these guidelines.

**Methods**: Percents were extracted from the abstracts of research articles published in 2017 in 23 selected journals. Percents were excluded if they referred to a statistical interval, typically a 95% confidence interval. Counts and percents were calculated for the number of percents using too few or too many decimal places, and these percents were compared between journals.

**Results**: The sample had over 43,000 percents from around 9,500 abstracts. Only 55% of the percents were presented according to the guidelines. The most common issue was using too many decimal places (33%), rather than too few (12%). There was a wide variation in presentation between journals, with the range of ideal presentation from a low of 53% ( *JAMA*) to a high of 80% ( *Lancet Planetary Health*).

**Conclusions**: Many percents did not adhere to the guidelines on using decimal places. Using the recommended number of decimal places would make papers easier to read and reduce the burden on readers, and potentially improve comprehension. It should be possible to provide automated feedback to authors on which numbers could be better presented.

## Introduction

“Everything should be made as simple as possible, but not simpler.” Albert Einstein (paraphrased).

Scientists read papers in order to keep up with the latest developments in their field and improve their research. However, the ever-increasing number of papers is placing greater demands on scientists’ time. In 2010 there were an estimated 75 trials and 11 systematic reviews published per day in the field of health and medicine ^1^, and by 2012 the number of systematic reviews had more than doubled to 26 per day ^2^. Papers have also become less readable over time, with an increase in the use of scientific jargon ^3^.

Poorly presented numbers can decrease readability and can distort or even hide important information. Statistical software packages show results to many decimal places, but this level of precision may be spurious, and authors may overcrowd a paper with numbers if they copy the results from software without considering what level of precision is appropriate. An often cited bad example are journal impact factors, which are reported to three decimal places when one or no decimal places would be enough to show the differences between journals ^4^.

Authors may also over-simplify numbers by rounding and losing important information. For example, a review of the gender bias in funding peer review reported in a results table that 20% of applicants were female in a study of 41,727 applications ^5^, so from these results we only know that the number of female applicants was somewhere between 8,137 and 8,554, a range of 417. To use these results in a meta-analysis it would be better to know the actual number of applicants. The large sample size in this example means that potentially useful information is lost by rounding the percent to an integer.

Authors must strike a balance between presenting numbers with too little or too much detail. The abstract and discussion are a summary of the findings, and here numbers can be rounded to make sentences easier to read. Numbers in the results section and tables can be presented with more detail, because they can be an accurate record of the data (e.g., for meta-analysis) and the reader is usually not expected to read every number in a table, especially a large table. Of course, tables can be made clearer by reducing unnecessary numbers, and so allowing the reader to easily comprehend the key information. There is a similar balance to consider when using acronyms in papers, as an overuse of acronyms can make a paper hard to understand because readers need to retrieve additional information, whereas using established acronyms can speed up reading.

There are guidelines by Cole ^6^ for presenting numerical data, including means, standard deviations, percentages and p-values. These guidelines are part of the wider EQUATOR guidelines for “Enhancing the QUAlity and Transparency Of health Research” http://www.equator-network.org/ ^7^. Cole’s guidelines for percentages are:

- Integers or one decimal place for values under 10%, e.g., 1.1%
- Integers for values above 10%, e.g., 22% not 22.2%
- One decimal place may be needed for values between 90% to 100% when 100% is a natural upper bound, for example the sensitivity of a test, e.g., 99.9% not 100%
- Use two or more decimal places only if the range of percents being compared is less than 0.1%, e.g., 50.50% versus 50.55%

There are also guidelines from journals and style guides. For example, the instructions to authors for the journal *Australian Zoologist* state that, “Numbers should have a reasonable and consistent number of decimal places.” The Australian style guide also recommends a consistent number of decimal places when comparing numbers, so “1.23 vs 4.56” not “1.23 vs 4.5” ^8^. The *Economist* style guide recommends, “resisting the precision of more than one decimal place, and generally favouring rounding off. Beware of phoney over-precision ^9^.”

It is not clear whether Cole’s guidelines on presenting numerical data are being adhered to, or if there is generally too little or too much rounding in published papers. An audit of 1,250 risk ratios and associated confidence intervals from the abstracts of *BMJ* papers between 2011 to 2013 found that one quarter of confidence intervals and an eighth of estimates could have been presented better ^10^. A single reviewer and editor of 53 biosciences articles found 29 (55%) reported too many decimal places for the mean ^11^.

This paper examines a large sample of percents in recent abstracts for multiple journals to examine how they are being presented.

## Methods

### Data extraction

I extracted percentages from abstracts available in *PubMed* using the “rentrez” R package (version 1.1.0) ^12^. Example abstracts are referred to using their *PubMed* ID number rather than citing the paper, and readers can find the paper’s details by putting the number into a *PubMed* search with the search term “[PMID]”.

I searched for papers in the following journals: *The BMJ*, *BMJ Open*, *Environmental Health Perspectives*, *F1000Research*, *JAMA*, *The Lancet*, *The Medical Journal of Australia*, *Nature*, *NEJM*, *PLOS ONE* and *PLOS Medicine*. These journals were selected to give a range of journals that publish articles in health and medicine, including some high profile journals and some large open access journals. To look at recent papers, I restricted the search to 2017. To focus on research papers, I restricted the search to article types of: Journal Article, Clinical Trial, Meta-Analysis, Review, Randomized Controlled Trial and Multicenter Study. The search returned 33,147 papers across 23 journals (searching for “ *The Lancet*” included all journals in the *Lancet* stable).

Despite the initial restriction on article type, the search results included non-research papers that had multiple types, e.g., a retraction of a clinical trial. Hence I excluded any papers that included an article type of: Biography, Conference, Comment, Corrected, Editorial, Erratum, Guideline, Historical, News, Lectures, Letter or Retraction.

**Figure 1.**
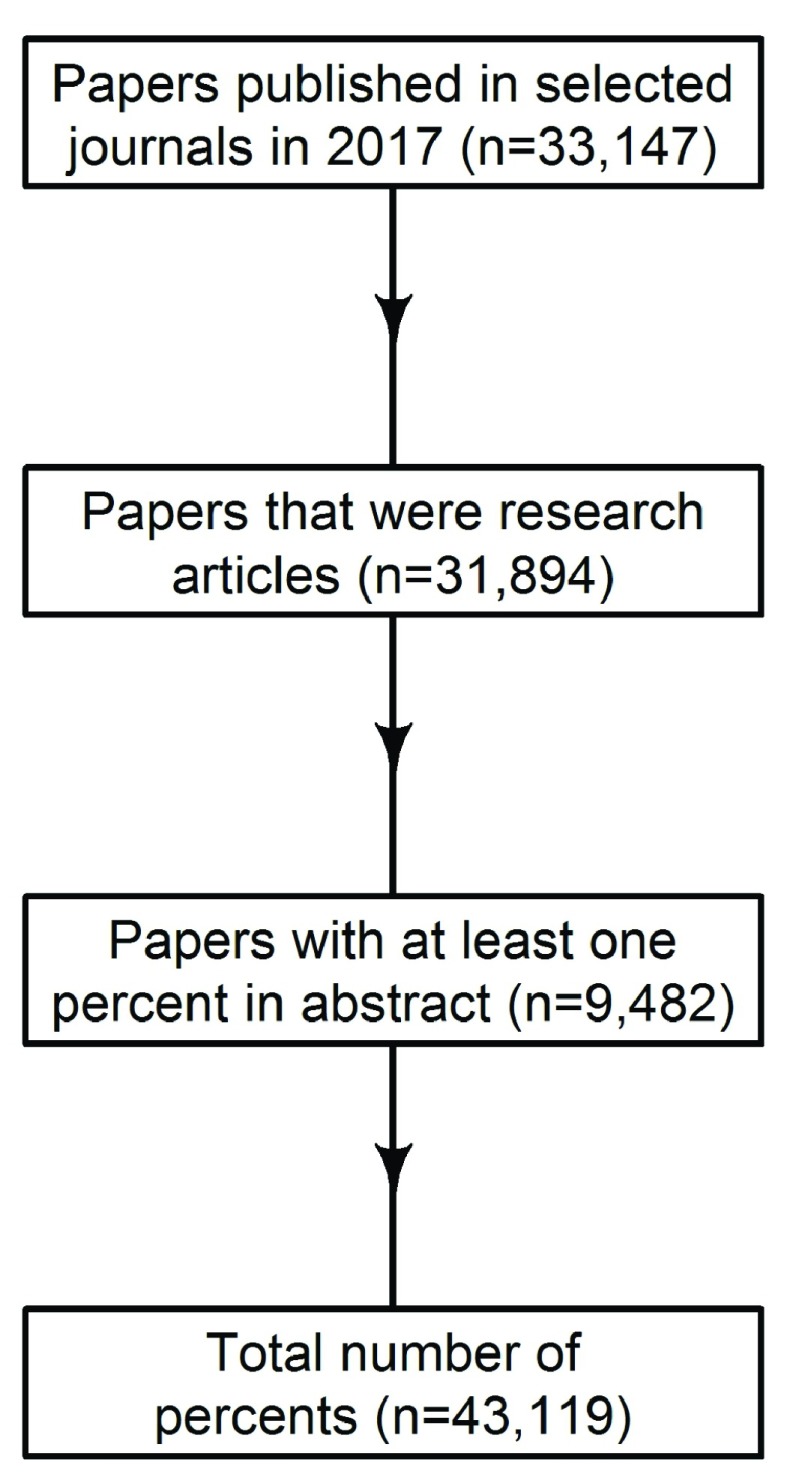
Flow diagram of included papers.

A flow diagram showing the selection of papers is in Figure 1.

I examined only percents because they are a widely used and important statistic, and are relatively easy to extract using text mining compared with other important statistics, such as the mean or rate ratio. I extracted all percentages from the text of the abstract by searching for all numbers suffixed with a “%”, with or without a preceding space. The key steps for extracting the percents from the abstract were:

1. Simplify the text by removing the “ *±*” symbol and other symbols such as non-separating spaces
2. Find all the percents
3. Exclude percents that refer to statistical intervals or statistical significance, e.g, “95% confidence interval”
4. Record the remaining percents as well as the number of decimal places and significant figures

The complete steps are detailed in the R code available here: https://github.com/agbarnett/decimal.places. Based on Cole’s guidelines ^6^, I defined the ideal number of decimal places as:

- 0 for percents between 10 and 90, and percents over 100
- 1 for percents between 0.1 and 10, and percents between 90 and 100, and percents of exactly 0
- 2 for percents under 0.1
- 3 for percents under 0.01
- 4 for percents under 0.001 but greater than 0

Preferably I would have also considered a greater number of ideal decimal places when the aim was to compare a small difference in two percents. For example, 10.5% compared with 10.6% in the same sentence ( *PubMed* ID 28949973) would both be considered as having one decimal place too many using the above guidelines, but the additional decimal place may be warranted if the small difference of 0.1% is clinically meaningful. However, accurately calculating a small difference of less than 0.1% requires all percents to be displayed using two or more decimal places. Ultimately I ignored this issue because it applied to so few abstracts.

I removed percents that referred to statistical intervals (e.g., “95% CI”) as these were labels not results. I searched for common interval percents of 80%, 90%, 95% and 99%. I combined these four percents with the words: “confidence interval”, “credible interval”, “Bayesian credible interval”, “uncertainty interval”, “prediction interval”, “posterior interval” and “range”. I included versions using capital and non-capital letters, and the standard acronyms including “CI” and “PI”. I also removed references to statistical significance percents using the common percents of 1%, 5% and 10% combined with the words: “significance”, “statistical significance” and “alpha level”.

I verified that the percents were correctly recorded for 50 randomly selected articles which contained 198 percents. There were no errors in the recorded percents, but there were 5 percents that were labels rather than results (e.g., “the prevalence of pretreatment NNRTI resistance was near WHO’s 10% threshold” *PubMed* ID 29198909), and there was an error with the ideal number of decimal places being 4 for a percent of 0% which led to a change in my ideal number of decimal places. There was also a “95% fixed kernel density estimator” which is a statistical interval and illustrates the difficulty of removing every type of statistical interval. I also checked the percentages for the abstract with the largest number of percents and the abstracts with the largest number of decimal places and significant figures. I also checked some abstracts that included percents of exactly 95% to check for any missing interval definitions. These checks led to additional definitions of intervals including the non-standard arrangements of “95% IC”, "CI 95%" ( *PubMed* ID 28228447) and the typo "uncertainly interval" ( *PubMed* ID 29171811).

I only extracted percents that were suffixed with the percent symbol. For example, the only extracted percent for the text “5–10%” or “5 to 10%” would be 10%. Any percents written in words were also not extracted. I also did not extract numbers immediately before the word “percent” or “per cent” as I assumed that these would be rare. I ignored the sign of the percent as I was primarily interested in presentation, so for example “–10%” was extracted as 10%. Similarly “ *<*10%” was extracted as 10%. I only used the abstracts, rather than the main text, because: 1) abstracts are freely available on *PubMed* for a wide range of journals, whereas the full text can only be easily text-mined for open access journals such as *PLOS ONE*, 2) the abstract is a summary of the results and so percentages should be presented according to Cole’s guidelines, whereas percents may be presented with more decimal places in the results in order to give an accurate and reusable record of the data.

### Statistical analysis

I calculated the difference between the observed number of decimal places and the ideal number as defined above. Because most differences were within *±*1, I categorised the data into: too few, just right, and too many. I plotted the three categories by journal. I estimated confidence intervals for the percents in these three categories using a Bayesian Multinomial Dirichlet model ^13^. The large sample size meant all confidence intervals had a width of 2% or less when using the complete sample, hence I did not present these intervals as they were not useful. The intervals are used to summarise the uncertainty for the results from journals. I did not adjust for the clustering of multiple percents within the same abstract.

In a sensitivity analysis I excluded percents that could be due to digit preferences, which were those with no decimal places that were a multiple of 10 as well as 75%. I also excluded percents between 90% and 100% because these may or may not have had a natural upper bound at 100%, and so it is difficult to automatically judge whether they should be presented with one or no decimal places. The data extraction and analyses were made using R (version 3.4.3) ^14^. All the data and code are available here: https://github.com/agbarnett/decimal.places.

## Results

**Figure 2.**
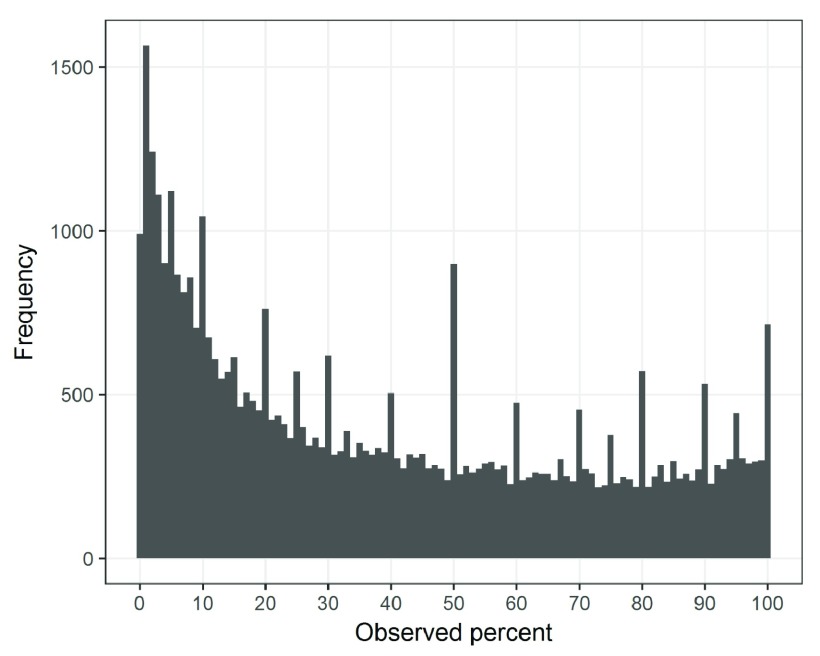
Histogram of all percents between 0% and 100% in 1% bins.

There were 43,119 percents from 9,482 abstracts. Over half the percents were from *PLOS ONE* ( Supplementary Table 1). The median number of percents per abstract was 3 with an inter-quartile range from 2 to 6. A histogram of all percents between 0 and 100 is shown in Figure 2, this excludes the 195 percents (0.05%) that were greater than 100%. There are spikes in the histogram at multiples of 10% and at 1%, 5%, 75% and 95%, these are likely due to digit preferences where percents have been rounded to commonly used values. To investigate this further I examined fifty randomly selected percents of exactly 50 (including 50.0 or 50.00) and found that 20 (40%) were results, with the remainder being thresholds (e.g., “Over 50% of elderly people die in acute hospital settings” *PubMed* ID 28526493) or rounded results (e.g., “We find that around 50% of liver genes are significantly zonated” *PubMed* ID 28166538).

The percent and number of percents meeting the guidelines are in Table 1. The recommended number of decimal places were used just over half the time. When the number decimal places differed from the guidelines, it was more likely to be too many decimals (33%) rather than too few (12%). Only 21% of abstracts (1,947 out of 9,482) used the ideal number of decimal places for every percent. After excluding the digit preference percents, as many of these were not results, the recommended number of decimal places were used just 50% of the time and the percent of time that too many decimal places were used increased to 40%.

**Table 1.** Percent and number of percents in abstracts where the guidelines on decimal places were used or not.

Decimal places	All percents % (number)	Excluding digit preferences % (number)
Too few	12 (4,981)	10 (3,574)
Just right	55 (23,872)	50 (17,408)
Too many	33 (14,266)	40 (14,047)
Total	100 (43,119)	100 (35,029)

An example where too many decimal places were used is, “True retentions of *α*-tocopherol in cooked foods were as follows: boiling (77.74-242.73%), baking (85.99-212.39%), stir-frying (83.12-957.08%), deep-frying (162.48-4214.53%)” ( *PubMed* ID 28459863).

An example where too few decimal places were used is, “263 [3%] of 8313 vs 158 [2%] of 8261” ( *PubMed* ID 29132879). As the numerators and denominators are given, we can recalculate the two percents using the recommended one decimal place, which are 3.2% and 1.9%, respectively, a difference of 1.3%. Without the reader working out these percents, the implied difference could be smaller than 0.1% because 3% could be as little as 2.5% (rounded up to 3%) and 2% could be as large as 2.4% (rounded down to 2%).

There were abstracts where the number of decimal places varied within the same sentence, for example, “pre-2010 vs post-2010 31.69% vs 64%” ( *PubMed* ID 29138196).

Some percents which I judged as having too few decimal places, were potentially harshly judged because the sentence aimed to give general percents, for example the following sentence probably did not need the percents to one decimal place, “it is a common, chronic condition, affecting 2–3% of the population in Europe and the USA and requiring 1–3% of health-care expenditure” ( *PubMed* ID 28460828).

Some percents with too few decimal places according to Cole’s guidelines were presented using a consistent number of decimal places, for example, “we noted reductions in genotypes 6 and 11 (from 12% [95% CI 6-21%], to 3% [1-7%]” ( *PubMed* ID 28460828); using the guidelines all the percents under 10% should have had one decimal place.

Some percents with too few decimal places were correctly presented with no decimal places because the sample size was under 100, for example, “with a specimen obtained at 13 to 15 months, in 1 of 25 (4%)” ( *PubMed* ID 26465681). I could not adjust for this correct presentation because I did not extract sample sizes.

### Results by journal

**Figure 3.**
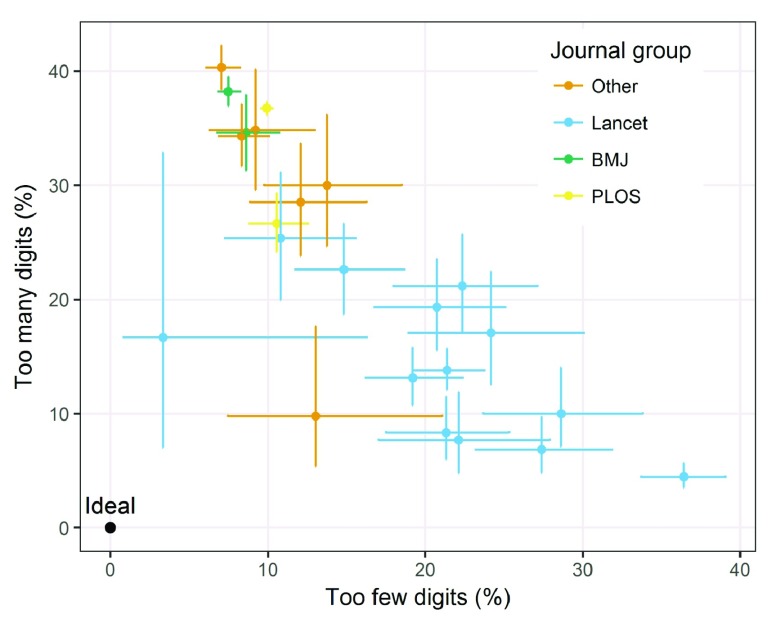
Scatter plot of the percent of times journals used too few and too many decimal places with 95% confidence intervals (horizontal and vertical lines).

There were large differences between some journals in the number of decimal places used ( Figure 3). There is some grouping of *Lancet* journals, which collectively leaned towards using too few decimal places. The two journals with results closest to the ideal were *Lancet Planetary Health* and *Nature*, although the sample size for *Lancet Planetary Health* is just 30 ( Supplementary Table 1). There was a negative correlation between using too few and too many decimal places, so journals that used too many decimals places were less likely to use too few, and vice versa.

Only two journals had specific guidelines about decimal places in their online instructions to authors ( Supplementary Table 2) and these both concerned not using decimal places where the sample size was under 100 (sensible advice which I did not consider here). Some instructions to authors did encourage the use of the EQUATOR guidelines, from where Cole’s guidelines for decimal places are available.

## Discussion

Numerical results are vitally important for quantitative research papers. Presenting numbers with too many decimal places can unnecessarily tax the reader and obscure important differences, whereas too much rounding makes it hard to compare results and can make differences appear too small. Overall, I found that only around half of all percents were presented according to the guidelines for decimal places, and the most common problem was using too many decimals. The overuse of decimals may stem from a belief that more numbers reflect greater accuracy. It is also likely that most researchers are not aware of the guidelines for presenting percents and other statistics.

The guidelines are not written in stone and good arguments can be made for not using them in some circumstances, for example, using no decimal places where all the percents are just above and below 10%, or where the differences are large enough to clearly show importance (e.g., a 1% versus 9% difference in mortality instead of 1.0% versus 9.0%). Hence the “around half” estimate for imperfect presentations found here likely overstates the problem. Additionally, there are far more serious mistakes that can be made with numbers, such as using the wrong data ^15^ or mislabelling statistics.

I found large differences between journals in the number of decimal places used. These differences could be due to editorial policy and also differences in the training and experience of the journals’ author cohorts. *Nature* had one of the best results in terms of ideal presentation, and they published relatively few papers, which may mean they have more time to edit papers for clarity and presentation. *PLOS ONE* had the most amount of papers in the sample and did relatively badly compared with the guidelines, perhaps because there is no time for editors to fix issues with presenting numbers given the large volume of papers and other important tasks, for example, checking for plagiarism and undeclared competing interests.

The difference in standards between journals likely adds to the confusion for authors about how to present numbers. Greater consistency and better presentation might be improved by having an automated checking procedure similar to the *statcheck* program that checks for errors in statistical reporting ^16^. This could be used to flag numbers that may need changing and could be part of an automated submission process for journals through online writing tools such as *Overleaf* ^17^. Automating the process would reduce the burden on journal staff.

I only examined percents, but it is likely that other statistics, such as means and risk ratios, are also being imperfectly presented. In fact, using percents may underestimate the problem of spurious precision because percents are almost always between –100 and 100, whereas means can take on a far wider range depending on the unit of measurement, and a wider range of numbers creates more opportunity for poor display. I only examined percents because these are the easiest numbers to automatically extract from text, thanks to the “%” suffix.

## Conclusions

Many percents in abstracts did not adhere to the guidelines on using decimal places. A more considered use of decimal places would increase readability and potentially improve comprehension.

## Data availability

The data referenced by this article are under copyright with the following copyright statement: Copyright: © 2018 Barnett AG

Data associated with the article are available under the terms of the Creative Commons Zero "No rights reserved" data waiver (CC0 1.0 Public domain dedication). 

All the data and code are available here: https://github.com/agbarnett/decimal.places.

Archived data and code as at time of publication: doi:10.5281/zenodo.1300056 ^18^.

## Supplementary material

Supplementary Table 1. Table of the percent of times journals used too few and too many decimal places according to the guidelines.

Supplementary Table 2. Instructions to authors about decimal places for percents from the selected journals.
