# Supplementary material for: Missing the point: are journals using the ideal number of decimal places?
Source: F1000Res. 2018 Aug 10;7:450. Originally published 2018 Apr 11. [Version 3] doi: 10.12688/f1000research.14488.3 (PMC6073092; doi:10.12688/f1000research.14488.3)
Supplement: Supplementary file 1 [file f1000research-7-17282-s0000.tgz › fddd59ac-ba9b-4ba4-8845-21571472bb6a.docx]

Table S1. Table of the percent of times journals used too few and too many decimal places according to the guidelines. The observed numbers are in brackets. Rows ordered by the percent “just right”.

| **Journal** | **Too few** | **Just right** | **Too many** | **Total** |
| --- | --- | --- | --- | --- |
| JAMA | 7.1% (158) | 53% (1179) | 40% (903) | 2240 |
| PLOS ONE | 9.9% (2513) | 53% (13490) | 37% (9295) | 25298 |
| BMJ Open | 7.5% (412) | 54% (2990) | 38% (2102) | 5504 |
| MJA | 9.2% (27) | 56% (164) | 35% (102) | 293 |
| EHP | 14% (33) | 56% (135) | 30% (72) | 240 |
| Lancet, HIV | 22% (76) | 56% (192) | 21% (72) | 340 |
| BMJ | 8.6% (68) | 57% (448) | 35% (273) | 789 |
| NEJM | 8.3% (94) | 57% (647) | 34% (387) | 1128 |
| Lancet, P | 24% (51) | 59% (124) | 17% (36) | 211 |
| Lancet, O | 36% (506) | 59% (821) | 4.5% (62) | 1389 |
| F1000 | 12% (36) | 59% (177) | 29% (85) | 298 |
| Lancet, D&E | 21% (73) | 60% (211) | 19% (68) | 352 |
| Lancet, RM | 29% (83) | 61% (178) | 10% (29) | 290 |
| Lancet, GH | 15% (59) | 63% (249) | 23% (90) | 398 |
| PLOS Med | 11% (112) | 63% (665) | 27% (282) | 1059 |
| Lancet, PH | 11% (23) | 64% (136) | 25% (54) | 213 |
| Lancet | 21% (279) | 65% (846) | 14% (180) | 1305 |
| Lancet, H | 27% (112) | 66% (269) | 6.8% (28) | 409 |
| Lancet, ID | 19% (120) | 68% (423) | 13% (82) | 625 |
| Lancet, N | 22% (46) | 70% (146) | 7.7% (16) | 208 |
| Lancet, G&H | 21% (87) | 70% (287) | 8.3% (34) | 408 |
| Nature | 13% (12) | 77% (71) | 9.8% (9) | 92 |
| Lancet, Planet | 3.3% (1) | 80% (24) | 17% (5) | 30 |
