# Supplementary material for: Missing the point: are journals using the ideal number of decimal places?
Source: F1000Res. 2018 Aug 10;7:450. Originally published 2018 Apr 11. [Version 3] doi: 10.12688/f1000research.14488.3 (PMC6073092; doi:10.12688/f1000research.14488.3)
Supplement: Supplementary file 2 [file f1000research-7-17282-s0001.tgz › b0b55ca1-5417-4126-876e-c8b96e11c2bc.docx]

**Table S2. Instructions to authors about decimal places for percents from the selected journals.**

| **Journal** | **Instructions to authors** |
| --- | --- |
| BMJ | None |
| BMJ Open | None |
| Environmental Health Perspectives | None |
| F1000Research | None |
| JAMA | “Do not use decimal places (ie, xx%, not xx.xx%) if the sample size is less than 100.” |
| Nature | None |
| PLOS Medicine | None |
| PLOS ONE | None |
| The Lancet* | None |
| The Medical Journal of Australia | “Do not use percentages if the denominator is < 100, and round up percentages to one decimal place for denominators of 100–1000, or two decimal places for denominators greater than 1000.” |
| The New England Journal of Medicine | None |

* All Lancet journals in the sample referred to the same author guidelines.

Search of web sites made on 6 April 2018.
