# Supplementary material for: Missing the point: are journals using the ideal number of decimal places?
Source: F1000Res. 2018 Aug 10;7:450. Originally published 2018 Apr 11. [Version 3] doi: 10.12688/f1000research.14488.3 (PMC6073092; doi:10.12688/f1000research.14488.3)
Supplement: Peer review history

# Peer review: Referee response for version 2

**DOI:** 10.5256/f1000research.16895.r36335

**Published:** 2018-08-01

**Authors:** David J. Winter

**Affiliations:** 1 Massey University, Palmerston North, New Zealand

This revision addresses the major issues associated with the code and manuscript, and the paper should thus be accepted. However, two minor issues associated with the manuscript have not been dealt with, and the author's reply to reviews does not explain this decision. To reiterate:

- The statement in the third paragraph of the introduction starting "Papers have been criticised for using too many decimal places..."  is still not supported by a citation (the cited paper is an example of a paper using high precision despite a small sample size, not a criticism of this practice). It should be simple to re-word the sentence or, if papers have been criticized for this practice, cite examples.
- it would be better to cite the paper describing rentrez, rather than the CRAN repository

These issues may seem trivial, and they should not hold up the acceptance of this manuscript, but I think they should be addressed in a final revision.

I have read this submission. I believe that I have an appropriate level of expertise to confirm that it is of an acceptable scientific standard.

## References

[1] WinterDJ: rentrez: an R package for the NCBI eUtils API. *The R Journal*.2017;9(2) :520-526

# Peer review: Referee response for version 1

**DOI:** 10.5256/f1000research.15773.r33075

**Published:** 2018-06-26

**Authors:** David J. Winter

**Affiliations:** 1 Massey University, Palmerston North, New Zealand

In this article, Barnett examines the precision with which percentages are reported in the abstracts of journals indexed in PubMed. The article does a good job of explaining the motivation and design of the research and putting its results into context. The data presented in the manuscript supports the major finding of the work (that percentages are frequently presented with more apparent-precision than is reasonable). 

 Because my own expertise relates more to the code used for this analysis than the proper presentation of statistics my review focuses on the scripts use to perform the analysis. The linked code repository is currently lacking a number of files required to perform the analysis. I am sure this is simply and oversight, but I cannot recommend this paper for acceptance until these files are included. In addition, the overall design of the analysis code, which contains hundreds of lines of R code without any user-defined functions and no "high-level" documentation of different sections of the scripts, makes it difficult to sport errors or apply this approach to other datasets. I detail these concerns below.

**Major issues with code**

 The files 'decimalplaces.R' and 'MultinomialCIsBayes.R' are required to perform these analyses but not included in the repository.

 Many of the datasets produced by this analysis are only saved in binary formats (excel spreadsheets or serialized R objects). Key data underlying the results presented in the paper should be made available in a plain-text format (e.g. csv for the tables).

 The README file for the github repository should contain:

 (a) brief description of the most important files included in the repository and their purpose (e.g. make.data.R, decimal.places.stats.Rmd) 

 (b) A list of R packages required to run this code 

 (c) Instructions on how a user can run these analyses (perhaps pointing out any steps that take a particularly long time, and how they might be skipped)

 (d) Links to this paper and the Zenodo archive associated with this repository.

 The file "make.data.R" would benefit from some high-level documentation explaining the motivations for each section of the file, and precisely what is being created by each code block. At present, the comments and the top of each block are very brief and difficult to interpret. For example, the comment # get meta data (loop through smaller numbers)" (appearing on line 101) has no obvious meaning to me.

**Minor issues with** **code**

 I suggest you avoid using the aliases T and F for TRUE and FALSE. These are not  reserved variables, and accidentally setting them to some other value can lead to unexpected results.

 The code often uses an idiom like 

 already.saved = T if(already.saved==F) {     #do something }

 I am not sure what purpose this serves, as the value is always hard-coded to TRUE. If the intend is to save users from re-running the data-fetching step it may make more sense to save the data to csv, then check for the existence of the data file before fetching fresh data.

 The variable "journals2" is defined by never used. 

 Line 146 has "for (a in 1:9000)", hard-coding the number of articles to 9000 (slightly fewer than the number included in the study).

 A lot of typing could be saved from variables like "ci.phrases" if abstracts where always converted to lower case before matching strings. This would both increase the readability of the code and make it less likely that typographical errors slip into the code.

**Minor issues with the manuscript.**

 The word "accuracy" is often used to describe the representation of percentages. I think "precision" is a better term for what is being described.

 In the introduction, the sentence starting "Papers have been criticised for using too many decimal places..."

 does not cite any criticism.

 Rentrez now has a paper to cite

 Winter, D. J. (2017) rentrez: an R package for the NCBI eUtils API ^1^.

I have read this submission. I believe that I have an appropriate level of expertise to confirm that it is of an acceptable scientific standard, however I have significant reservations, as outlined above.

## References

[1] WinterDJ: rentrez: an R package for the NCBI eUtils API. *The R Journal*.2017;9(2) :520-526

# Response

**Published:** 2018-06-30

**Authors:** Adrian Barnett

**Affiliations:** Queensland University of Technology, Australia

Thanks for your useful comments on the code and documentation thereof.

- The files 'decimalplaces.R' and 'MultinomialCIsBayes.R' have now been added to github.
- All the key RData files have now also been provided as CSV or tab-delimited files.
- The README file in github is more detailed.
- I’ve added sections to the make.data.R code and improved the comments.
- I've used TRUE/FALSE in place of T/F
- As suggested, I’ve now checked for the existence of the RData files.
- The variable "journals2" was used as a time saver. The many data were created for two sets of journals (‘journals’ and ‘journals2’).
- The loop up to 9000 was added because there were sometimes breaks in the online connection to pubmed. I’ve used the full loop and added a warning in the code about this issue.
- I kept the text in upper case because I originally also wanted to find sentences and used the pattern of: full-stop then space then upper-case letter, to define a new sentence. Changing to lower case included some non-sentence breaks for odd character strings such as compounds or labels from genomics. The idea was to compare percentages in the same sentence, but I did not use this in the paper.

# Peer review: Referee response for version 1

**DOI:** 10.5256/f1000research.15773.r33072

**Published:** 2018-04-23

**Authors:** Tim J. Cole

**Affiliations:** 1 UCL Great Ormond Street Institute of Child Health, London, UK

Barnett's article tests the adherence of papers in 23 journals to Cole's guidelines on the numerical presentation of percentages. As the author of the guidelines I acknowledge a competing interest. The paper is well designed, executed and reported, and it has a clear message - many papers give percentages to excessive precision. I have a few minor suggestions for improving it.

 1. The term "accuracy" used several times in the Introduction and elsewhere should more correctly be "precision".

 2. Did the algorithm for identifying percents allow for the possibility of the % appearing preceded by a space?

 3. Looking at the R code I note the long lists of alternative spellings. First converting all text to lower case would have substantially simplified this process.

 4. The spikes in Figure 1 are interesting. The author suggests they represent digit preference, which may be true, but I would have thought it worth formally checking some of them to see.

 5. The paper starts with an apt quotation from Einstein. I wanted to draw the author’s attention to another apt quotation, from Gauss, which appears as a response to my original Archives of Disease in Childhood paper here. It is "Lack of mathematical education does not become more evident than by excessive precision in numerical calculation." Carl Friedrich Gauss (1777-1855).

 Tim Cole

I have read this submission. I believe that I have an appropriate level of expertise to confirm that it is of an acceptable scientific standard.

# Response

**Published:** 2018-06-30

**Authors:** Adrian Barnett

**Affiliations:** Queensland University of Technology, Australia

Thanks for your useful comments on the paper. My answers to your numbered questions are:

1. Agreed and changed in the Introduction and Discussion.
2. Yes, a preceding space was allowed. I have now added this to the Methods.
3. I kept the text in upper case because I originally also wanted to find sentences and used the pattern of: full-stop then space then upper-case letter, to define a new sentence. Changing to lower case included some non-sentence breaks for odd character strings such as compounds or labels from genomics. The idea was to compare percentages in the same sentence, but I did not use this in the paper.
4. This comment was based on my informal checks made whilst compiling the data. As a more formal check I examined fifty randomly selected percents of exactly 50 and found that only 20 were actual results, with the rest being rounded results or thresholds. I’ve added some additional text to the paper on this.
5. Thank you for this quote. Excessive decimal places have been a problem for some time and this paper will hopefully help to draw attention to it again.
